# Targeting HCG18 counteracts ferroptosis resistance via blocking the miR-30a-5p/RRM2/GSS pathway in hepatocellular carcinoma

**DOI:** 10.7150/ijbs.104127

**Published:** 2025-03-21

**Authors:** Tian Zhan, Yawei Liu, Shuoke Duan, Chen Lu, Heng Jia, Ming Jin, Jie Li, Xinru Du, Sizheng Sun, Yuan Li, Jianping Zhang

**Affiliations:** 1 Department of General Surgery, The Affiliated Cancer Hospital of Nanjing Medical University, Nanjing, 210009, China.; 2 Department of General Surgery, The Second Affiliated Hospital of Nanjing Medical University, Nanjing, 210000, China.; 3 Department of Gastroenterology, the Affiliated Suzhou Hospital of Nanjing Medical University, Suzhou, 215031, China.; 4 The Key Laboratory of Modern Toxicology, Ministry of Education, School of Public Health, Nanjing Medical University, Nanjing, 211166, China.; 5 Department of General Surgery, Sir Run Run Hospital of Nanjing Medical University, Nanjing, 211100, China.; 6 Department of Oncology, The Second Affiliated Hospital of Nanjing Medical University, Nanjing, 210000, China.; 7 Xiamen Humanity Hospital, Fujian Medical University, Fujian, 350122, China.

**Keywords:** HCG18, ferroptosis therapy, RRM2, GSS, hepatocellular carcinoma

## Abstract

**Background:** Finding effective strategies and novel targets for reversing drug resistance is one of the major frontiers in hepatocellular carcinoma (HCC) research. Ferroptosis is participate in the malignant progression and drug resistance of HCC. However, the underlying molecular mechanisms remail largely uninvestigated.

**Methods:** HCC cell lines and xenografted nude mice were used as experimental models. Biological functions were investigated by various molecular biology experiments. An HCC population was used to reveal clinical significance.

**Results:** In our study, HCG18 and RRM2 was found to be associated with unfavorable prognosis. HCG18 regulates RRM2 expression through competitively binding to miR-30a-5p, consequently impacting ferroptosis. RRM2 directly regulated GSS to increase GSH synthesis. The colony formation assay demonstrated that overexpression of HCG18 inhibited erastin-induced cell death. In addition, *in vivo* experiments have also confirmed that HCG18 can inhibit ferroptosis by regulating the expression of RRM2, thereby promoting HCC proliferation.

**Conclusion:** Our study discovered a novel lncRNA HCG18, as a “switch-like” molecule of the axis of miR-30a-5p/RRM2/GSS, confers resistance to ferroptosis and holds promise as a potential target for ferroptosis-dependent therapy.

## 1. Introduction

Liver cancer is a prevalent and fatal disease that poses a considerable threat to global public health, characterized by its highly malignant nature and ranking as the third most significant cause of cancer-related deaths 1. HCC stands out as the prevailing form of liver cancer 2. With the advancement in medical care and surgical techniques, the long-term survival rate of HCC patients has also been raised to a certain degree. However, over 85% patients are usually diagnosed at an advanced stage, leading to miss optimal timing of surgery 3. Currently, chemotherapy and immunotherapy represent the foremost therapeutic modalities for patients with advanced stages, but their efficacy remains suboptimal 3, 4. Hence, further research to explore better therapy methods to patients with the advanced HCC are necessary.

Ferroptosis is a unique type of programmed cell death that relies on iron and is distinguished by the abnormal buildup of lipoperoxides, differentiating it from other forms of cellular demise 3. Iron serves a crucial function as a pro-oxidant in ferroptosis, engaging in reactions with hydrogen peroxide to produce toxic lipid ROS, ultimately culminating in cellular death 5. Then ROS is transformed into nontoxic lipid alcohols under the action of glutathione peroxidase 4 (GPX4) and GSH 6. Emerging studies have demonstrated that Erastin and RSL3 can reduce the production of GSH through inhibiting system Xc^-^ and inactivating GPX4, respectively, to induce cell ferroptosis 7. Additionally, multiple research studies have indicated a close correlation between ferroptosis and various diseases, including malignant tumors, thereby suggesting its potential as a pivotal mechanism for tumor suppression 6, 8-10.

LncRNAs lacked protein-coding potential play vital roles in tumorigenesis, metastasis and drug resistance 11,12. Recent research findings have indicated that certain lncRNAs participate in ferroptosis by acting as competing endogenous RNAs (ceRNAs). NEAT1 facilitates ferroptosis through controlling miR-362-3p/MIOX regulatory pathway in HCC 13, while PVT1 serves as a ceRNA to suppress miR-214, thereby promoting ferroptosis in brain ischemia/reperfusion injury 14. The dysregulation of HCG18, a crucial lncRNA, has been observed in various cancer types and is prominently expressed in diverse tumor tissues 15. For example, HCG18 promote ovarian carcinoma cells proliferation, migration, and epithelial-mesenchymal transition (EMT) through upregulating TRAF4/TRAF5 expression 16. Moreover, HCG18 overexpression facilitates the advancement of lung adenocarcinoma by modulating the axis involving miR-34a-5p and HMMR 17. However, the involvement of HCG18 in ferroptosis and its underlying regulatory mechanisms remain largely uninvestigated.

RRM2 is a member of the ribonucleoside diphosphate reductase small chain family, which plays important roles in cancer proliferation 18. The protein expression of RRM1 remains relatively stable in cells, whereas the protein expression of RRM2 exhibits dynamic changes in response to stimulation 19. RRM2 is considered a crucial tumor regulator and a potential tumor biomarker 20. In addition, a study has also shown that RRM2 acted as an anti-ferroptotic role through sustaining GSH synthesis in liver cancer 21. At present, the upstream and downstream molecular regulation of RRM2 in ferroptosis remains incompletely elucidated. Here, we have discovered that HCG18 can regulate RRM2 expression by competitive binding miR-30a-5p, thereby exerting suppressive effects of ferroptosis in HCC. Our study elucidates the function and regulatory mechanism of HCG18 in ferroptosis and provides a novel therapeutic strategy for HCC.

## 2. Materials and methods

### 2.1. Research data sources

Transcriptomes (GSE69164, GSE77509, GSE76903, TCGA-LIHC) were downloaded from the GEO and TCGA databases. Ferroptosis-related genes were collected from the FerrDb database. Tumor tissues from 150 HCC patients and paracancer tissues from 40 HCC patients were procured at Second Affiliated Hospital of Nanjing Medical University between 2018 and 2022 following curative surgery. Additionally, complete survival information and clinical data of patients were collected. The results are presented in Supplementary Table 1. Ethical approval for the utilization of human tissue sections was provided by the ethics committee of the Second Affiliated Hospital of Nanjing Medical University.

### 2.2. Identification of DEGs

Following the R package instructions, the R package “DESeq2” was employed to analyze transcriptome data from the TCGA database and the R package “limma” was employed to analyze transcriptome data from the GEO database. The selection criteria of differentially expressed genes (DEGs) were described in our previous article 22.

### 2.3 Construction of ferroptosis-related ceRNAs network

All differentially expressed mRNAs (DEMs) and genes from the FerrDb database were intersected to obtain the ferroptosis-related DEMs. The "clusterProfiler" package was used to perform Gene Ontology (GO) and Kyoto Encyclopedia of Genes and Genomes (KEGG) enrichment analysis on differentially expressed genes associated with ferroptosis, aiming to investigate their potential roles and pathways in HCC. The miTarBase database was utilized for the prediction of miRNA-mRNA binding sites. Subsequently, taking the intersection between target miRNAs and DEMIs to obtain ferroptosis-related DEMis. LncBase and starBase database were applied to forecast lncRNA-miRNA interactions. Then, a set of ferroptosis-related DELs was gained by intersecting two sets of target lncRNAs. The Cytoscape software (version 3.6.1) was used to visualize a potential ceRNA regulatory network based on the interactions of DEGs related to ferroptosis. Additionally, survival analysis and Pearson correlation analysis were employed to determine the regulatory axis, with selection criteria set at p-value < 0.05 and a correlation coefficient > 0.40.

### 2.4 Cell lines and cell culture

The HepG2 cell line (NO. HB-8065) was obtained from the American Type Culture Collection (ATCC; USA), while the Huh7 cell line (NO. JCRB0403) was purchased from the Japanese Collection of Research Bioresources cell bank (JCRB; Tokyo, Japan). Both HCC cell lines were cultured in Dulbecco's modified Eagle's medium (DMEM; Gibco, USA) supplemented with 10% fetal bovine serum (FBS; HyCyte, China) at 37℃ under 5% CO2 atmosphere. 1% penicillin-streptomycin (Thermo, USA) was added to the medium to avoid cell contamination.

### 2.5 Cell transfection

Transfection experiment was performed when cells were at 70 to 90% confluence. Lipofectamine 3000 (Invitrogen) and Liposomal Transfection Reagent (Hieff Trans) were used in cell transfection based on the manufacturer's instructions. The miR-30-5p mimics, inhibitor, small interfering RNA negative control (siNC), siHCG18 and siRRM2 were constructed from Gene Pharma (Shanghai, China). The HCG18, RRM2 and GSS overexpression plasmids were obtained from Genomeditech (Shanghai, China). The siRNA or plasmid sequences were showed in Supplementary Table 2. All constructs were verified by PCR.

### 2.6 qRT-PCR

The trizol method was utilized for the extraction of total RNAs from the treated cells or tissues, while the reverse transcription kit (Vazyme, Nanjing, China) was employed to synthesize cDNA. The qRT-PCR experiment was performed by using the ChamQ SYBR Green qPCR Master Mix kit (Vazyme, Nanjing, China). The primers used in our study are provided in Supplementary Table 2 and include HCG18, RRM2, miR-30a-5p, GSS as well as glyceraldehyde 3-phosphate dehydrogenase (GAPDH) and U6. GAPDH and U6 were utilized as endogenous controls.

### 2.7 Colony formation assay

After a period of 48 hours following transfection, the cells were subjected to erastin or RSL3 treatment for 24 hours. Subsequently, the cells were digested, counted and reseeded in appropriate numbers into 6-well plates. Following a two-week culture period, the cells were treated with methanol for 15 minutes to fix them, followed by staining with 0.4% crystal violet for 30 minutes. The quantification of colony numbers was employed to response the ability of cell proliferation.

### 2.8 Immunoblotting (IB)

The RIPA lysis buffer (Beyotime, China) containing 1% PMSF or a phosphatase inhibitor was utilized for cellular lysis. The BCA assay kit (Beyotime, China) was utilized to detected protein concentration. The proteins underwent separation using SDS-PAGE and were subsequently transferred onto PVDF membranes. After a 2-hour blocking period with 5% BSA, the membranes were subjected to primary antibody incubation at 4℃ for a duration of 12 to 16 hours, which the secondary antibodies were incubated with them at room temperature for approximately 2 hours. Antibodies used in this study were listed in Supplementary Table 3.

### 2.9 Cell viability assay

The cell activity was conducted by employing cell counting kit-8 (CCK-8; C0038, Beyotime). Cells were reseeded into 96-well plates at a density of 5000 cells per well and subsequently treated with erastin or RSL3 for a duration of 24 hours. Then, each well added to 10 μl of CCK-8 solution and incubated an additional period of 2 hours. The absorbance at 450nm was measured to assess the cell activity.

### 2.10 Lipid ROS detection

The content of lipid ROS was quantified using flow cytometry with BODIPYTM 581/591 C11 (D3861, Thermo). Cells were enumerated and seeded in six-well plates at a density of 2 ×10^5^ cells per well, followed by treatment with erastin or RSL3 for 24 hours. Subsequently, the cells were washed twice with PBS and stained with 5 µM BODIPYTM 581/591 C11. After incubation for 15 minutes, the cells were washed at least three times and resuspended them in fresh PBS. Number of fluorescence cells in each sample were detected and analyzed by the FlowJo software.

### 2.11 Intracellular GSH detection assay

The concentration of GSH was determined using the GSH detection assay kit (ab112132, Abcam). First, cells were counted and reseeded in 24-well plates with 2 ×10^4^ cells in per well and overnight incubation. Then, cells were treated with erastin or RSL3 for 24 hours, and then dyed with Thiol Green Dye for 30 min. Fluorescence microscope was used to take pictures, and the quantitative analysis of luminescent cells' mean fluorescence intensity was conducted utilizing the ImageJ software.

### 2.12 Iron assay

The concentration of ferrous iron (Fe^2+^) in each sample was quantified using the iron assay kit (TC1015, LEAGENE). The cells were grown in 10 cm^2^ dishes and exposed to erastin or RSL3 for a duration of 24 hours at 90% confluence. The protein was extracted and the protein concentration was detected for subsequent calculations. The 96-well plates were washed with dilute hydrochloric acid and deionized water. Afterwards, the mixed liquor with protein sample to be tested and corresponding test reagents was added to a 96-well plate according to the instructions in the user manual. After standing for 15 minutes, the measurement of absorbance at 562 nm was conducted using a microplate reader within a time frame of one hour. The amount of Fe^2+^ in each sample was calculated, zeroed with the blank well and normalized with the iron standard (2 μg/ml) well.

### 2.13 Dual-luciferase reporter gene assay

The Luciferase Reporter Assay kit (ab287865, Abcam) was utilized to perform the luciferase reporter assay based on the manufacture's instruction. To generate the HCG18 and RRM2-MT, we employed a gene mutation technique to alter the binding site (AUGUUUAC) to (UACAAAUG), followed by cloning the corresponding full-length or mutant form complementary DNA (cDNA) into the PGL3-CMV-LUC-MCS vector. The constructs were verified by sequencing. The HCC cells were co-transfected with either the HCG18-WT or MT plasmid, along with either NC or miR-30a-5p mimics. Meanwhile, the RRM2-WT or MT plasmid and either NC or miR-30a-5p mimics were co-transfected into HCC cells. Subsequently, cell lysis was performed using cell lysis buffer and relative light unit (RLU) of per sample was detected.

### 2.14 Xenotransplantation experiment

In our study, the BALB/c nude mice were obtained from the SLRC Laboratory Animal Center (Shanghai, China). All animal-related experimental procedures were performed in compliance with the guidelines endorsed by the Institutional Animal Care and Use Committee at Nanjing Medical University. For the xenograft model, the human Huh7 cells (1×10^7^ cells) stably overexpressing HCG18 was administered via subcutaneous injection in the right axilla of per nude mouse. Erastin (40 mg/kg) was used to treat mice via intraperitoneal injection every 3 days 23. The miR-30a-5p agomir was obtained from RiBoBio Co. Ltd (Guangzhou, China. No. miR-40000128-4-5). HCG18-siRNA, RRM2-siRNA, and miR-30a-5p agomir was intratumoral injection every 3 days (100 μl siRNA, 100 nM; 50 μl of miRNA-agomir, 60 nM). Tumor volumes were recorded every 3 days using the following formula: volume =1/2 (width^2^ × length). After a duration of 21 days, the mice were euthanized and subcutaneous tumors were surgically extracted for further study.

### 2.15 Lipid peroxidation malondialdehyde (MDA) assay

The content of MDA within the cell was detected using the Lipid Peroxidation MDA Assay Kit (Beyotime, China) following the instructions provided by the manufacturer. The proteins were extracted from mouse tumor tissues by using IP cell lysis buffer (P0013, Beyotime). The absorbance was measured at 532nm.

### 2.16 Immunohistochemistry (IHC)

The immunostaining of RRM2 or Ki67 was performed on xenograft mouse tumor tissues and human HCC tissues that had been fixed in formalin and embedded in paraffin. Antibodies used in this study were listed in Supplementary Table 3. The quantitative analysis of the gray value for each figure was conducted utilizing the ImageJ software 24.

### 2.17 Co-immunoprecipitation (Co-IP)

The RRM2 plasmid with 3×Flag label and GSS plasmid with 3×HA label were co-transfected into cells. The primary antibody was added to the cell lysates and incubated overnight at 4 ℃, followed by incubation with A/G magnetic beads (Thermo). The collected beads were utilized to perform IB analysis. Antibodies used in this study were listed in Supplementary Table 3.

### 2.18 Validation in populations

In order to further validate the clinical value of HCG18 and RRM2, human HCC tissues were subjected to qPCR for HCG18 or immuno-stained for RRM2. The ImageJ software was utilized to perform a quantitative analysis of the gray value in each section. According to the median value (for HCG18) or to median gray value (for RRM2), patients were categorized into HCG18 high expression and HCG18 low expression, or into RRM2 high expression and RRM2 low expression, respectively. Survival analysis and clinical relevance analysis (tumor stage, age and gender) were performed using R statistical software.

### 2.19 Statistical analysis

All experimental data were statistically analyzed using Graphpad Prism (version 8.0) and R statistical software (version 3.6.1). Statistical data were presented as mean ± standard deviation (SD), with each experiment conducted a minimum of three times. The standard t-test was employed to compare differences between the two groups, considering a p-value < 0.05 as statistically significant.

## 3. Results

### 3.1 Revealing a HCG18-miR-30a-5p-RRM2 axis

**Figure 1:**
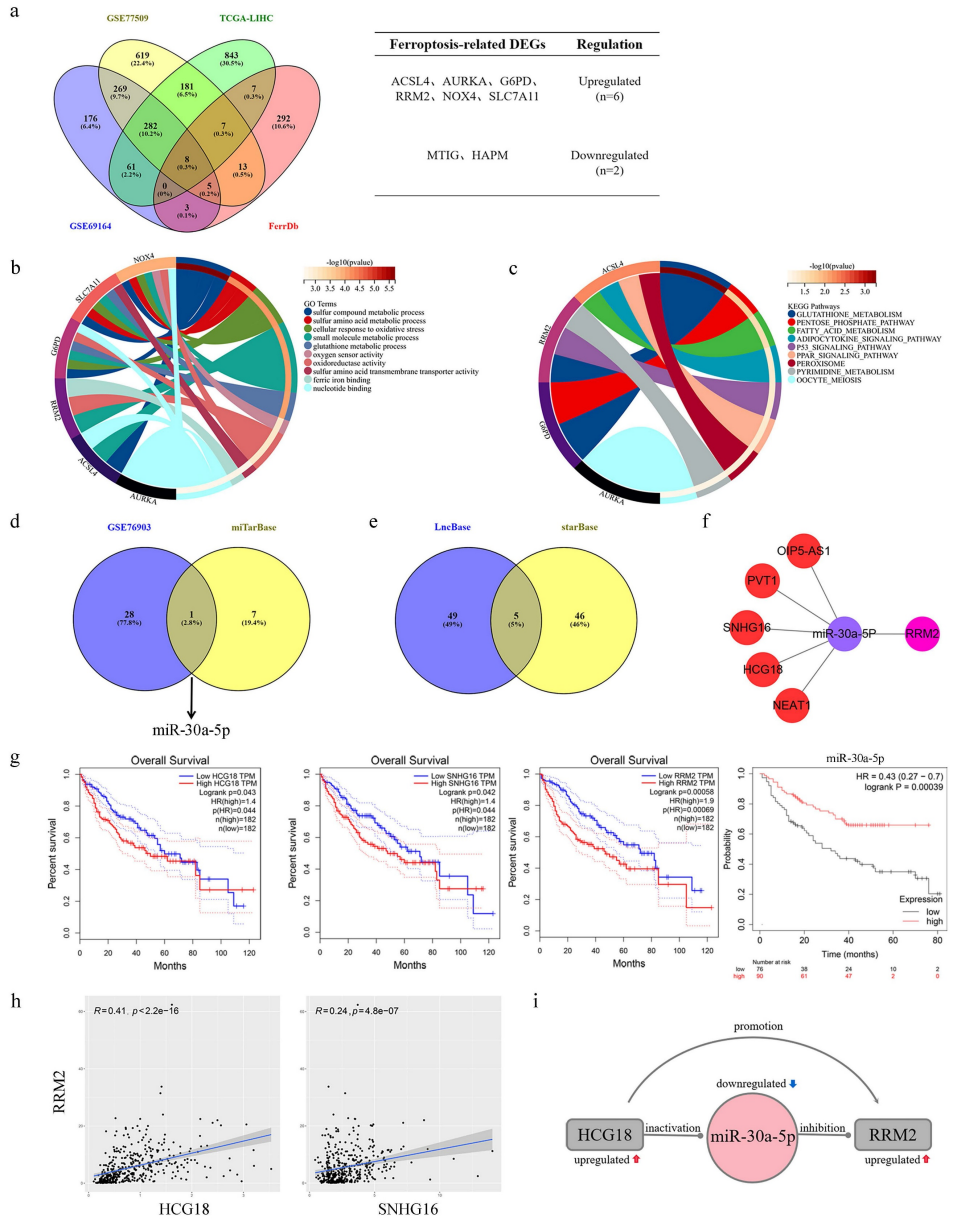
Identification of HCG18-miR-30a-5p-RRM2 axis in HCC. **a** (Left) Venn map showed the overlapping mRNAs in significantly differentially expressed mRNAs from GSE69164, GSE77509, TCGA-LIHC and ferroptosis-related mRNAs from FerrDb. (Right) Intersection gene list and gene expression. **b** GO and **c** KEGG enrichment analysis of ferroptosis-related differentially expressed genes in HCC. **d** Venn map showed miR-30a-5p was the only overlapping miRNA between significantly differentially expressed miRNAs in GSE76903 and predicted miRNAs in miTarBase. **e** Venn map showed the overlapping lncRNAs interacting with miR-30a-5p were predicted by LncBase and starBase. **f** Construction of ceRNA regulatory network based on 5 lncRNAs, a miR-30a-5p and a mRNA. **g** Kaplan-Meier survival analysis of HCG18, SNHG16, RRM2 and miR-30a-5p in HCC. **h** Pearson correlation analysis of RRM2-HCG18 and RRM2-SNHG16. **i** The potential regulatory relationship between HCG18, miR-30a-5p and RRM2.

A combined total of 804, 1384, and 1389 DEGs were identified from the GSE69164, GSE77509, and TCGA-LIHC datasets correspondingly (the results were as described previously 22). Then, the intersection of these DEGs and collected genes related to ferroptosis yielded a set of eight DEGs associated with ferroptosis in HCC (Fig. 1a). The main roles of these genes involved cellular response to oxidativestress, oxidoreductase activity, glutathione metabolic process and oxygen sensor activity, and glutathione metabolism was major regulatory pathway according to the results of GO and KEGG analysis (Fig. 1b-c). Afterwards, 29 DEMis and 8 targeted miRNAs were gained from GSE76903 dataset and miTarBase database, respectively. The intersection of the two sets of miRNAs was showed in Fig. 1d, that miR-30a-5p was the only ferroptosis-related DEMi in HCC. Based on miR-30a-5p, a set of five targeted lncRNAs (OIP5-AS1, PVT1, SNHG16, HCG18 and NEAT1) were obtained from the intersection of LncBase and starBase databases (Fig. 1e). Finally, a ceRNAs network was constructed from 5 lncRNAs, a miRNA, and a mRNA (Fig. 1f). To determine the clinical value of the ceRNAs network in HCC, survival analysis was conducted, revealing that HCC patients with high expression of HCG18, SNHG16 or RRM2 had poor prognosis, whereas those with high expression of miR-30a-5p demonstrated a longer survival period (Fig. 1g). Due to no significant correlation for survival time, OIP5-AS1, PVT1 and NEAT1were excluded from ceRNAs network (Supplementary Fig. 1). Further, the results of correlation analysis indicated that HCG18 (R=0.41) had a more significant positive correlation with RRM2 than SNHG16 (R=0.24) (Fig. 1h). Therefore, we revealed a HCG18-miR-30a-5p-RRM2 regulatory axis associated with ferroptosis in hepatocellular carcinoma, and the interactions were depicted in Fig. 1i.

### 3.2 Clinical significance of HCG18 and RRM2

**Figure 2:**
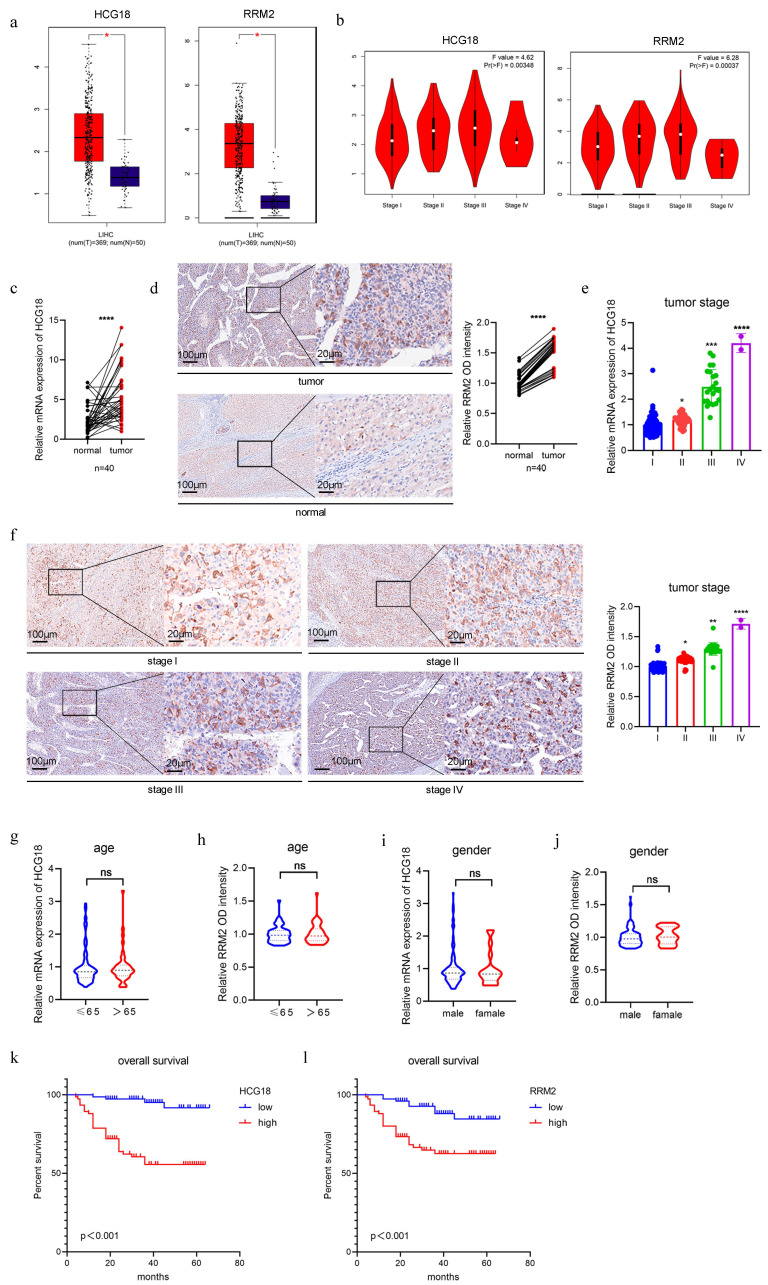
Clinical significance of HCG18 and RRM2. **a** TCGA database was used to analyze the expression levels of HCG18 and RRM2 in HCC tissues and normal liver tissues.** b** TCGA database was used to analyze the correlation between the expression of HCG18 and RRM2 and tumor stage. **c-d** The relative expression of HCG18 and RRM2 in HCC tissues and adjacent normal tissues of 40 cases. **e-j** The relative expression of HCG18 and RRM2 in different tumor stages, age and gender of 150 cases.** k-l** Kaplan-Meier survival analysis of HCG18 and RRM2 in 150 HCC cases. The scale bar is 100µm and the magnification scale bar is 20µm. *P < 0.05, **P < 0.01, ***P < 0.001, ****P < 0.0001; ns, not significant.

In general, genes with high expression levels in tumor tissues or cells significantly contributes to tumor progression and therapy 25. Therefore, we mined the TCGA database and identified that HCG18 and RRM2 were significantly expressed in HCC tissues (Fig. 2a). Furthermore, their expression levels demonstrated a strong correlation with tumor stage (Fig. 2b). Then we collected tumor tissues from 150 HCC patients (including normal adjacent tissues in 40 cases), along with their corresponding clinical data (tumor stage, age and gender). As shown in Fig. 2c and 2d, significantly elevated expressions of HCG18 and RRM2 in HCC tissues were observed compared to adjacent non-cancerous tissues. Furthermore, a positive correlation was found between HCG18 or RRM2 expression and tumor stage (Fig. 2e and 2f). Nevertheless, no significant association was found between HCG18 or RRM2 expression and either age or gender (Fig. 2g to 2j). Subsequently, patients were stratified into two cohorts based on the expression levels of HCG18 or RRM2, with the median value serving as the cutoff point. Patients exhibiting high expression of HCG18 or RRM2 were found to have significantly shorter survival time compared to those with low expression, as determined through Kaplan-Meier survival analysis (Fig. 2k and 2l). Collectively, these results further reveled the clinical significance of HCG18 and RRM2.

### 3.3 HCG18 inhibited ferroptosis in HCC cells

**Figure 3:**
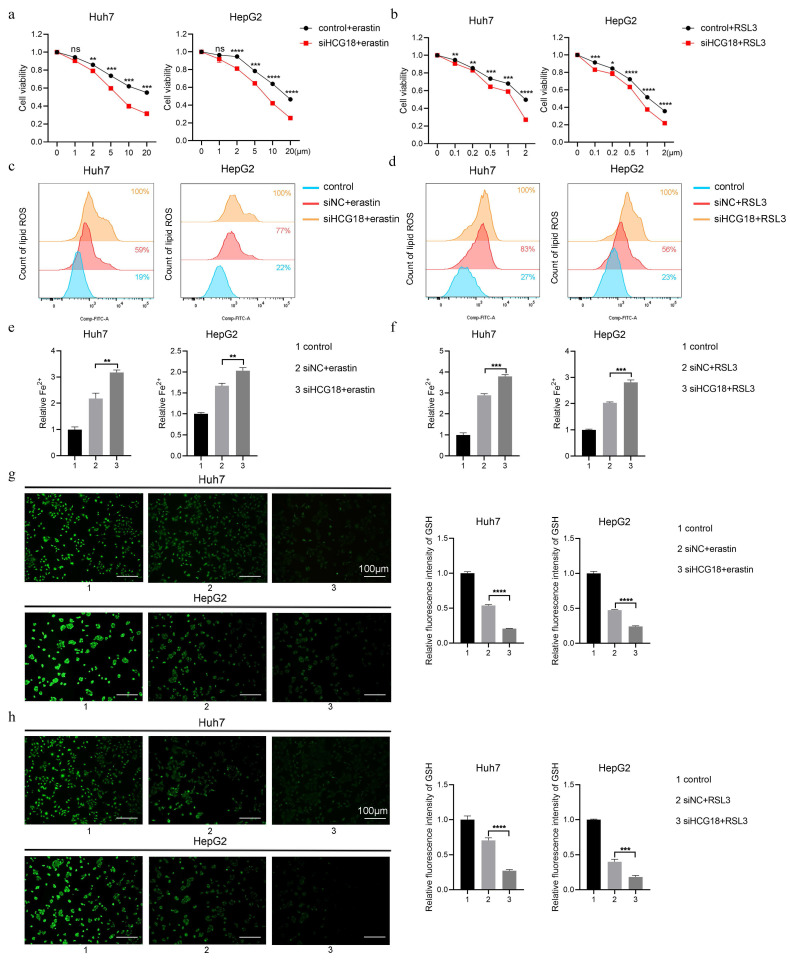
Knockdown of HCG18 promotes ferroptosis in HCC cells. **a-b** CCK-8 assay was used to measure the cell viability, **c-d** flow cytometry was used to measure lipid ROS level, **e-f** iron detection assay was used to detected intracellular Fe^2+^, and **g-h** GSH detection assay was used to measure intracellular GSH concentration in control and siHCG18 HCC cells treated with erastin or RSL3 for 24 h. Control group represents non-treated cells. The scale bar is 100µm. *P < 0.05, **P < 0.01, ***P < 0.001, ****P < 0.0001; ns, not significant.

**Figure 4:**
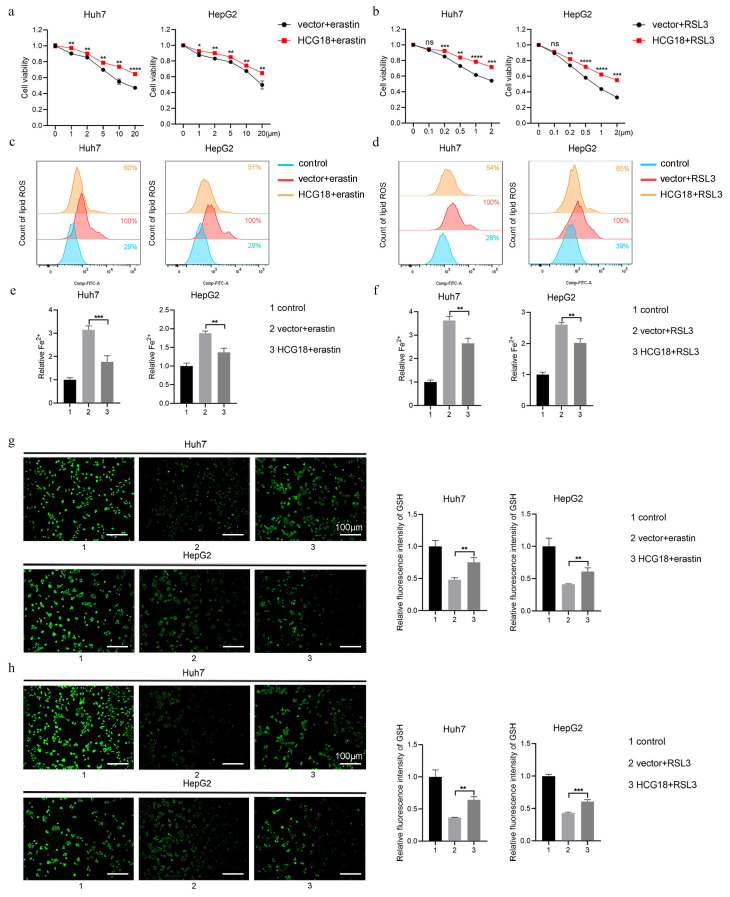
Overexpression of HCG18 inhibits ferroptosis in HCC cells. **a-b** CCK-8 assay was used to measure the cell viability, **c-d** flow cytometry was used to measure lipid ROS level, **e-f** iron detection assay was used to detected intracellular Fe^2+^, and **g-h** GSH detection assay was used to measure intracellular GSH concentration in vector and HCG18 HCC cells treated with erastin or RSL3 for 24 h. Control group represents non-treated cells. The scale bar is 100µm. *P < 0.05, **P < 0.01, ***P < 0.001, ****P < 0.0001; ns, not significant.

To clarify the impact of HCG18 on ferroptosis, Huh7 and HepG2 cell lines with manipulated levels of HCG18 were subjected to treatment with ferroptosis inducers. As shown in Fig. 3a and 3b, silencing of HCG18 resulted in a significant enhancement of cell death induced by erastin and RSL3. Next, due to the change of lipid ROS and Fe^2+^ are closely related to the ferroptosis process, and GSH is a pivotal endogenous antioxidant in the body 26, we measured the expression levels to further clarify the relationship between HCG18 and ferroptosis. The results manifested that silencing of HCG18 significantly promoted the accumulation of intracellular lipid ROS and Fe^2+^, while reducing the content of intracellular GSH (Fig. 3c to 3h). Conversely, we observed a significant reduction in erastin- and RSL3-induced cell death by overexpressing HCG18 (Fig. 4a and 4b). In addition, the overexpression of HCG18 led to a significant reduction of lipid ROS and Fe^2+^, while simultaneously elevating cellular GSH concentration (Fig. 4c to 4h). Cumulatively, the findings suggested that HCG18 had inhibitory effects on ferroptosis.

### 3.4 HCG18 regulated ferroptosis by impacting RRM2

**Figure 5:**
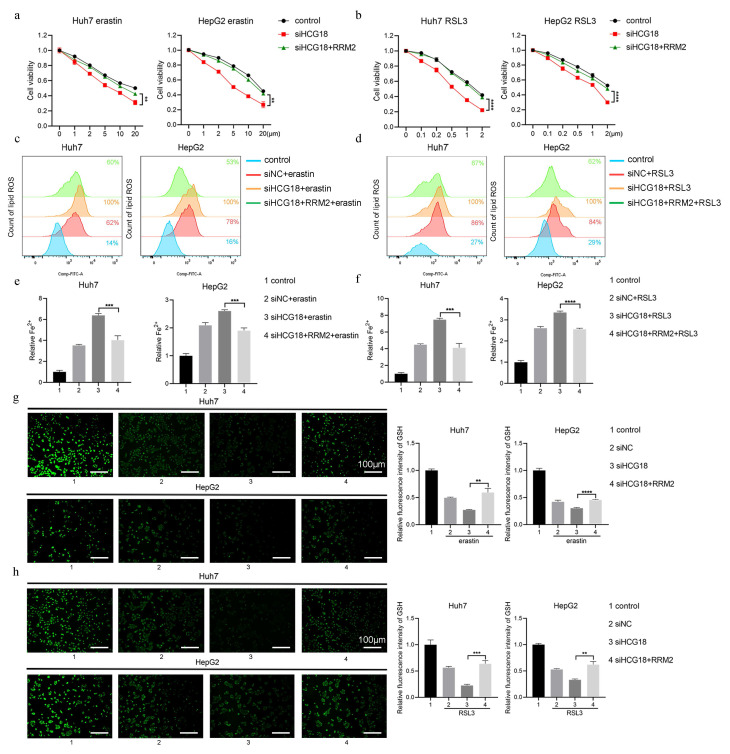
HCG18 regulates ferroptosis by impacting RRM2. **a-b** CCK-8 assay was used to measure the cell viability, **c-d** flow cytometry was used to measure lipid ROS level, **e-f** iron detection assay was used to detected intracellular Fe^2+^, and **g-h** GSH detection assay was used to measure intracellular GSH concentration in HCC cells transfected with indicated constructs and treated with erastin or RSL3 for 24 h. Control group represents non-transfected cells. The scale bar is 100µm. **P < 0.01, ***P < 0.001, ****P < 0.0001.

To deeply explore the underlying mechanism by which HCG8 regulates ferroptosis, RRM2, as a predicted target gene of HCG18, was overexpressed in erastin- and RSL3-induced cells. The results depicted in Fig. 5a and 5b demonstrate that enhanced RRM2 expression counteracted the facilitative impact of HCG18 deficiency on cell death induced by erastin and RSL3. In HCG18-silenced cells, intracellular lipid ROS and Fe^2+^ content were rescued upon the RRM2 overexpression (Fig. 5c to 5f). Similarly, the ferroptotic consumption of GSH significantly reduced by promoting the expression of RRM2 in HCG18 knockdown cells (Fig. 5g and 5h). In combination, these findings indicated that HCG18 indirectly facilitated the occurrence of ferroptosis through regulating the RRM2 expression.

### 3.5 miR-30a-5p was involved in the regulation of RRM2 by HCG18

**Figure 6:**
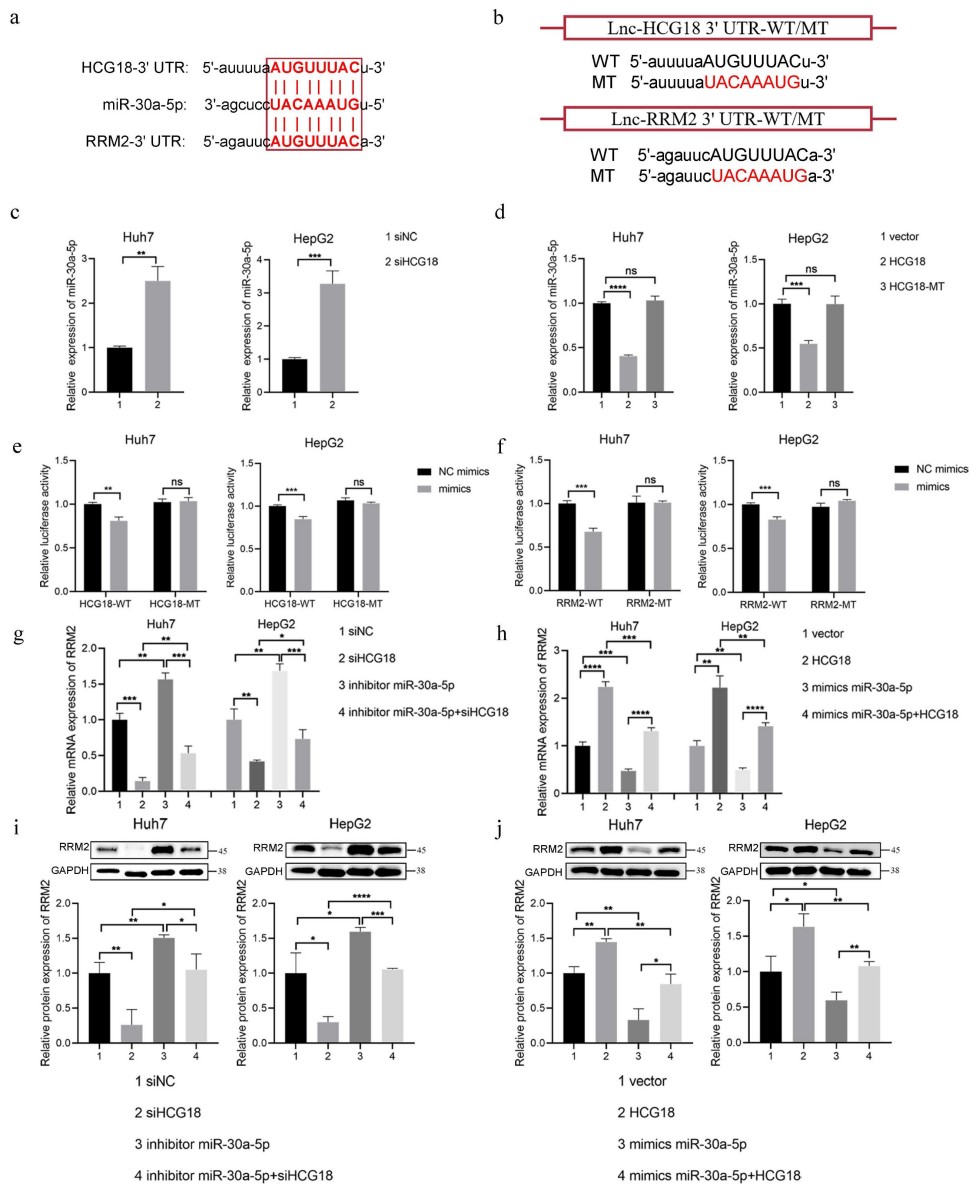
miR-30a-5p is involved in the regulation of RRM2 by HCG18. **a** StarBase database was used to predict the potential binding site for miR-30a-5p within HCG18 and RRM2.** b** Construction of wild type and mutant sequences of HCG18 and RRM2 3' UTR. **c** The relative expression levels of miR-30a-5p in siNC and siHCG18 HCC cells. **d** The relative expression levels of miR-30a-5p in vector, HCG18 and HCG18-MT HCC cells. **e** Luciferase reporter assay was used to valid the regulatory relationship between HCG18 and miR-30a-5p in HCC cells. **f** Luciferase reporter assay was used to valid the regulatory relationship between RRM2 and miR-30a-5p in HCC cells.** g-j** qRT-PCR and western blotting analysis were used to detected the relative expression level of RRM2 mRNA and protein in HCC cells transfected with indicated constructs. GAPDH was used as a loading control. *P < 0.05, **P < 0.01, ***P < 0.001, ****P < 0.0001; ns, not significant.

The mir-30a-5p plays crucial roles as a miRNA in cell proliferation and apoptosis 27. Based on the starBase platform, we found the possible binding sequences for miR-30a-5p within HCG18 and RRM2 (Fig. 6a). In order to validate the predictions made by bioinformatics analysis, we constructed HCG18 and RRM2-WT/MT plasmids to performed luciferase reporter gene assay in Huh7 and HepG2 cell lines (Fig. 6b). As shown in Fig. 6c, miR-30a-5p expression was markedly increased through knocking down HCG18. Conversely, overexpression of HCG18 markedly inhibited miR-30a-5p expression in HCC cells, but no significant alteration in miR-30a-5p expression upon transfected with HCG18-MT (Fig. 6d), which confirms that HCG18 competitively regulates miR-30a-5p expression. Furthermore, as depicted in Fig. 6e, the luciferase activity of HCG18 with wild-type sequence was notably diminished upon overexpression of miR-30a-5p, whereas no significant effect was observed on the mutant sequence. The result indicates that HCG18 directly sponge miR-30a-5p. Similarly, luciferase activity was markedly suppressed in cells harboring the RRM2-WT plasmid, while no significant alteration in the RRM2-MT cells, suggesting that miR-30a-5p directly binds to RRM2 (Fig. 6f). To find additional evidence of the involvement of miR-30a-5p in the reciprocal regulation between HCG18 and RRM2, we assessed RRM2 expression by PCR and western blotting analysis. As depicted in Fig. 6g to 6j, the downregulation of HCG18 was observed to impede RRM2 expression. But when miR-30a-5p was repressed, an increase in RRM2 expression was detected, which could be counteracted by reducing the levels of HCG18. On the contrary, miR-30a-5p overexpression significantly inhibited RRM2 expression, and this inhibitory effect was rescued by HCG18 overexpression. These findings suggested that HCG18 facilitated RRM2 expression through competitively binding miR-30a-5p.

### 3.6 miR-30a-5p promoted ferroptosis by regulating RRM2

**Figure 7:**
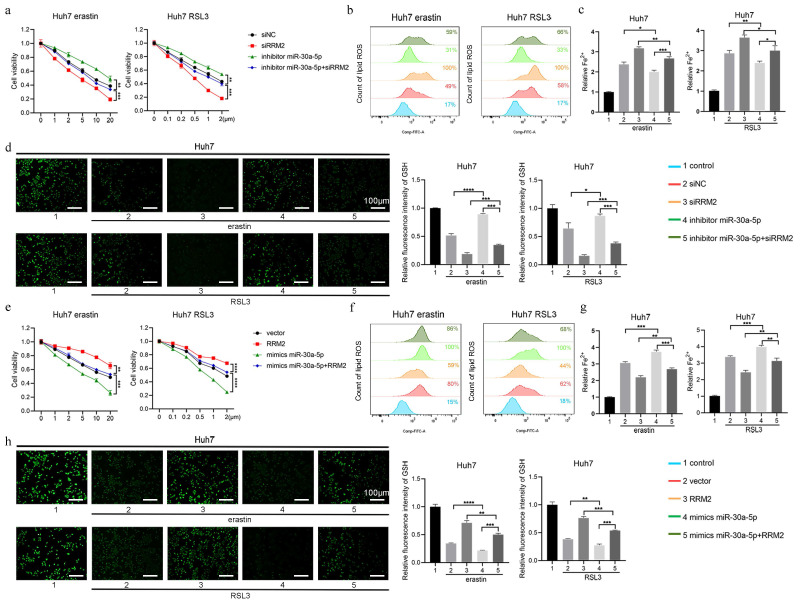
miR-30a-5p promotes ferroptosis by regulating RRM2. Huh7 cells transfected with indicated constructs were treated with erastin or RSL3 for 24 h, **a, e** CCK-8 assay was used to measure the cell viability, **b, f** flow cytometry was used to measure lipid ROS level, **c, g** iron detection assay was used to detected intracellular Fe^2+^, and **d, h** GSH detection assay was used to measure intracellular GSH concentration. Control group represents non-transfected cells. The scale bar is 100µm. *P < 0.05, **P < 0.01, ***P < 0.001, ****P < 0.0001.

To confirm whether miR-30a-5p regulated ferroptosis by impacting RRM2, the rescue experiment was performed in Huh7 cell line. As depicted in Fig. 7a, knockdown of miR-30a-5p markedly impeded cell death induced by erastin and RSL3, which was counteracted by downregulating the expression of RRM2. Similarly, the intracellular content of lipid ROS and Fe^2+^ markedly reduced in Huh7 cells containing miR-30a-5p inhibitor, which were restored upon inhibition of RRM2 (Fig. 7b and 7c). Meanwhile, knockdown of miR-30a-5p inhibited erastin- and RSL3-induced GSH depletion and the inhibitory effect was removed due to the downregulation of RRM2 (Fig. 7d). Furthermore, miR-30a-5p overexpression was found to notably enhance cell death induced by erastin and RSL3, while concurrently augmenting lipid ROS and Fe^2+^ accumulation and depleting GSH levels; however, overexpression of RRM2 resulted in inhibiting ferroptosis induced by miR-30a-5p (Fig. 7e to 7h), indicating that miR-30a-5p promoted ferroptosis through regulating RRM2 expression.

### 3.7 RRM2 directly regulated GSS to increase GSH synthesis

**Figure 8:**
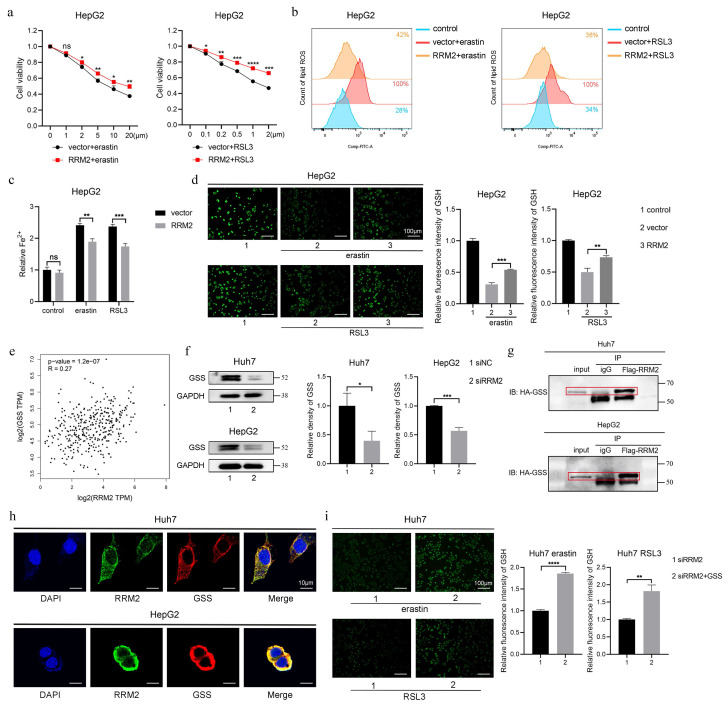
RRM2 directly regulated GSS to increase GSH synthesis. HepG2 cells transfected with vector or RRM2 were treated with erastin or RSL3 for 24 h, **a** CCK-8 assay was used to measure the cell viability, **b** flow cytometry was used to measure lipid ROS level, **c** iron detection assay was used to detected intracellular Fe^2+^, and **d** GSH detection assay was used to measure intracellular GSH concentration. Control group represents non-transfected cells. The scale bar is 100µm. **e** TCGA database was used to analyze the correlation between GSS and RRM2 in HCC. **f** Western Blotting analysis was used to detected the relative expression level of GSS protein in siNC and siRRM2 HCC cells. GAPDH was used as a loading control. **g-h** CO-IP and IF analysis were used to valid the interaction between RRM2 and GSS in HCC cells. The scale bar is 10µm. **i** GSH detection assay was used to measure intracellular GSH concentration in siRRM2 and siRRM2+GSS Huh7 cells treated with erastin or RSL3 for 24 h. The scale bar is 100µm. *P < 0.05, **P < 0.01, ***P < 0.001, ****P < 0.0001.

As mentioned above, RRM2 as a inhibitory gene for ferroptosis prevented cell death, production of lipid ROS and Fe^2+^, and depletion of GSH in Huh7 cells treated with erastin and RSL3. The inhibitory role was consistent with that in HepG2 cells (Fig. 8a to 8d), which manifest that RRM2 participate in iron and GSH metabolism. To further screen the target where RRM2 influences ferroptosis in HCC, we performed correlation analysis between RRM2 and key enzymes (CBS, CTH, GSS and GPX4) involved in GSH synthesis and metabolism using gene expression profiling interactive analysis (GEPIA) website 28-30. As shown in Fig. 8e and Supplementary Fig. 2, GSS as the only enzyme significantly associated with RRM2 was used to further study. Subsequently, we found that GSS expression was markedly suppressed in Huh7 and HepG2 cells containing siRRM2 (Fig. 8f). To confirm whether there was an interaction of RRM2 and GSS, we performed the Co-IP and confocal experiments in Huh7 and HepG2 cells. As expected, the results showed that RRM2 and GSS bind to each other, with main binding location being in the cytoplasm (Fig. 8g and 8h). In addition, as depicted in Fig. 8i, the overexpression of GSS effectively mitigated the promoting effect induced by siRRM2 on GSH consumption in ferroptosis. In summary, these evidences indicated that RRM2 partially played a role in ferroptosis by regulating GSS expression to modulate GSH levels.

### 3.8 Roles of HCG18 in erastin-induced ferroptosis *in vivo*

The modulation of HCG18 expression level is expected to influence the anti-tumor activity of erastin, as indicated by the observed effects on ferroptosis. Hence, we constructed a HuH-7 cell line with stable overexpression of HCG18 by a lentiviral vector (LV-HCG18) to investigate the impact of HCG18 on anticancer efficacy of erastin *in vivo*. The colony formation assay demonstrated that the proliferative capacity of the Huh7 cells treated by erastin was significantly reduced and overexpression of HCG18 inhibited erastin-induced cell death (Supplementary Fig. 3a).

**Figure 9:**
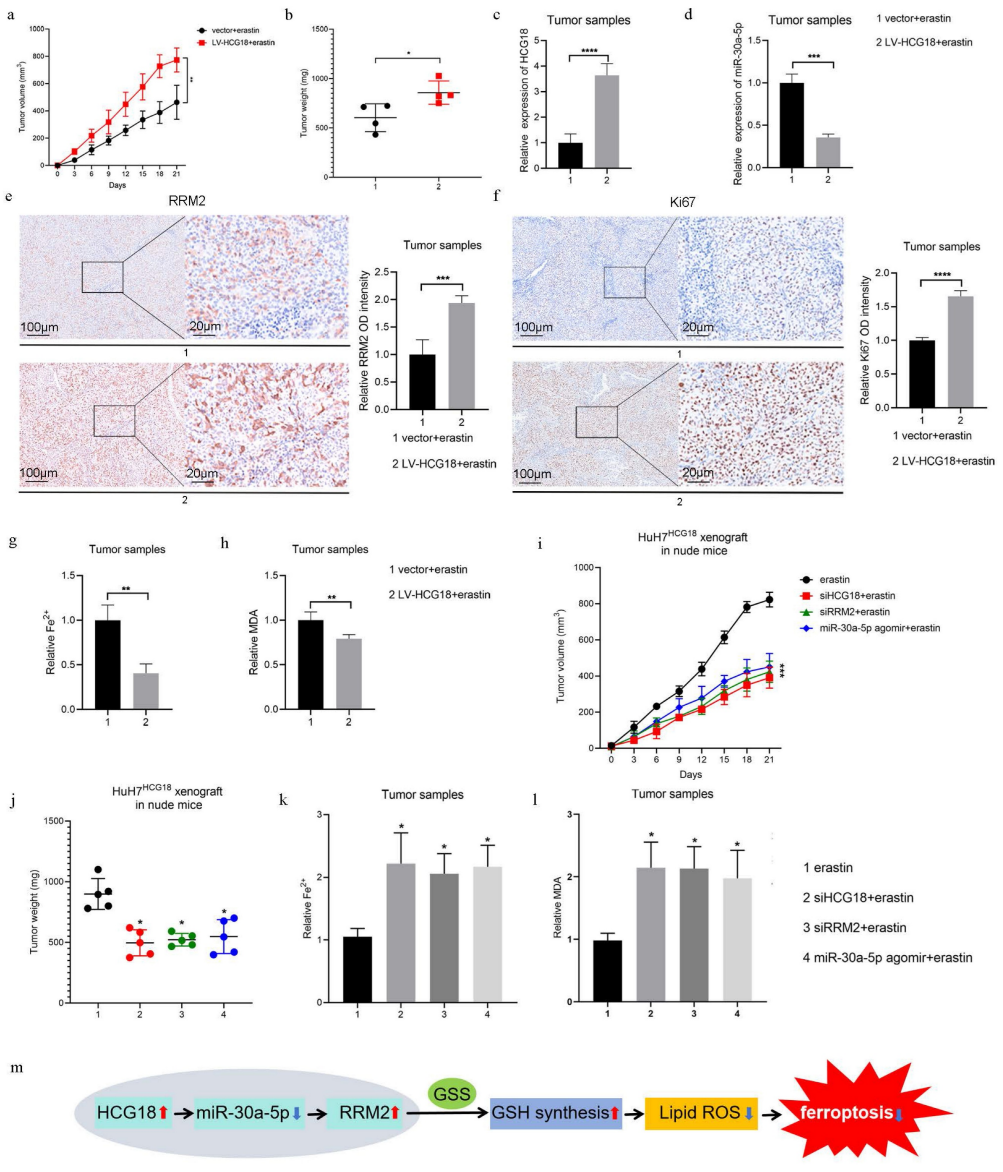
Roles of HCG18 in erastin-induced ferroptosis *in vivo*. **a** The change of subcutaneous tumor volume in nude mice after injecting Huh7 cells stably transfected with vector or LV-HCG18 over time. **b** Comparison of subcutaneous tumor weight in nude mice after injecting Huh7 cells stably transfected with vector or LV-HCG18. **c-f** The expression of HCG18, miR-30a-5p, RRM2 and Ki-67 in subcutaneous tumor tissues with HCG18 overexpression. The scale bar is 100µm and the magnification scale bar is 20µm. **g** Iron detection assay was used to detected Fe^2+^, and **h** Lipid Peroxidation MDA Assay was used to detected MDA content of subcutaneous tumor tissues in nude mice after injecting Huh7 cells stably transfected with vector or LV-HCG18.** i** The change of subcutaneous tumor volume in nude mice treated with erastin in the presence or absence of siHCG18, siRRM2, or miR-30a-5p agomir over time. **j** Comparison of subcutaneous tumor weight in nude mice treated with erastin in the presence or absence of siHCG18, siRRM2, or miR-30a-5p agomir.** k-l** The content of Fe^2+^ and MDA in subcutaneous tumor treated with erastin in the presence or absence of siHCG18, siRRM2, or miR-30a-5p agomir. **m** Schematic diagram of the regulatory mechanism of HCG18. *P < 0.05, **P < 0.01, ***P < 0.001, ****P < 0.0001.

Besides, consistent with previous studies 31, 32, we found that enhanced expression of HCG18 facilitated the growth of Huh7 cells, suggesting that HCG18 plays important roles in HCC (Supplementary Fig. 3a). Next, we injected the Huh7 cells stably overexpressing HCG18 into the right axilla of nude mice and treated with erastin every 3 days to induce ferroptosis (Supplementary Fig. 3b). During the process of subcutaneous tumor transplantation in nude mice, tumor growth was monitored at 3-day intervals over a period of 3 weeks. We observed that HCG18 overexpression resulted in a significant acceleration of tumor growth over time, with consistently larger tumor volumes (Fig. 9a and 9b). The result of PCR assay showed that the expression of HCG18 in the LV-HCG18+erastin group was significantly higher than that in the control vector group (Fig. 9c). We observed a significant decrease in miR-30a-5p expression and a significant increase in RRM2 expression in tumor tissues with HCG18 overexpression (Fig. 9d and 9e). To further verity the influence of HCG18 on tumor proliferation *in vivo*, we performed IHC on Ki67. As anticipated, the staining for Ki67 was markedly increased due to overexpression of HCG18 (Fig. 9f). In contrast to the control vector group, the overexpression of HCG18 markedly reduced the increase in Fe^2+^ and MDA induced by erastin in tumor tissues (Fig. 9g and 9h). Finally, we re-injected the Huh7 cells stably overexpressing HCG18 into the right axilla of nude mice and treated with erastin every 3 days in the presence or absence of siHCG18, siRRM2, or miR-30a-5p agomir. As shown in Fig. 9i to 9l, inhibition of HCG18 or RRM2, or activation of miR-30a-5p significantly decreased the tumor growth, but markedly increased the Fe^2+^ and MDA induced by erastin in tumor tissues. These results demonstrated that the HCG18/miR-30a-5p/MMR2 inhibited the anti-tumor activity of ferroptosis induced by erastin *in vivo*.

## 4. Discussion

The term "ferroptosis", initially proposed in 2012, denotes a regulated type of cell death induced by iron-dependent lipid peroxidation and ROS 33. In terms of morphology, biochemistry and genetics, this form of cell death exhibits distinct characteristics from apoptosis, necrosis, and autophagy 34. The primary features of ferroptosis encompass increased lipid oxidative stress and intracellular accumulation of labile iron ions 35. Due to its close association with various diseases such as cancer, metabolic disorders, neurological diseases and kidney injury, research on ferroptosis has emerged as a prominent and focal area of investigation in recent years.

Some recent studies suggest that epigenetic modification play important roles in the regulation of HCC 36. In our present study, we provided a novel HCG18/miR-30a-5p/RRM2/GSS pathway, regulating the ferroptosis resistance. LncRNAs play an important role in the advancement of cancer through their ability to control downstream gene expression and influence diverse biological processes. In recent years, numerous studies have been dedicated to investigating the regulatory correlation between lncRNA and ferroptosis. LncRNA BDNF-AS induces ferroptosis in peritoneal metastasis of gastric cancer through modulation of VDAC3 ubiquitination 37. Repression of lncRNA MALAT1 enhances the susceptibility of endometriosis to erastin-induced ferroptosis by regulating the miR-145-5p/MUC1 pathway 38. In prostate cancer, the inhibition of ferroptosis is facilitated by lncRNA OIP5-AS1 through its interaction with the miR-128-3p/SLC7A11 pathway 39. Hence, as research on the association between lncRNAs and ferroptosis advances, lncRNAs are expected to emerge as significant targets for tumor therapy. In our study, a significant downregulation of HCG18 was observed during induced ferroptosis. HCG18, as a competitive binder for miR-30a-5p, attenuated the susceptibility of HCC cells towards ferroptosis treatment by regulating the expression of RRM2.

GSH is a crucial antioxidant, safeguarding cells from harm caused by lipid peroxide 40. Through the utilization of GSH, ferroptosis is counteracted due to the conversion of lipid peroxides into their respective alcohols 41. GSS is a pivotal enzyme in the production of GSH from glutamate, cysteine, and glycine 42. The findings of a recent study indicate that the decreased activity of GSS can result in an elevation of lipid ROS levels within HepG2 cells 43. RRM2, a crucial enzyme, is accountable for the vital transformation of ribonucleotides to deoxyribonucleotides, ranking among the highly expressed enzymes identified in cancer 44.

The upregulation of RRM2 has been demonstrated to enhance the efficiency of DNA damage repair and replication, resulting in a reduced susceptibility to gemcitabine chemotherapy 45. Consistent with our study, elevated levels of RRM2 have been linked to unfavorable outcomes in multiple types of malignancies including liver cancer, pancreatic tumors and stomach cancer 46-48. Here, we observed that GSS was subject to strict regulation by RRM2 and knockdown of RRM2 resulted in decreased expression of GSS. Meanwhile, the functions of RRM2 in decreasing GSH metabolism and maintaining intracellular GSH levels was dependent on GSS. In addition, we also found that RRM2 can inhibit ferroptosis by accelerating iron metabolism, suggesting that RRM2 is an important regulator gene involving multiple metabolic pathways in ferroptosis.

Recent studies have suggested that HCG18 plays a pivotal role in various cancers by influence different miRNA. Overexpression of HCG18 promotes osteosarcoma cell proliferation by augmenting aerobic glycolysis through regulation of the miR-365a-3p/PGK1 axis, suggesting that HCG18 may serve as a promising therapeutic target for osteosarcoma intervention 49. lncRNA HCG18 can upregulate NOTCH1 by competitively binding to miR-34c-5p, thereby inhibiting bladder cancer 50. Here, we found that HCG18 could inhibit ferroptosis by miR-30a-3p/RRM2 pathway in HCC. The findings from these studies show that HCG18 plays a pivotal role as a lncRNA in orchestrating the interplay between apoptosis, autophagy, and ferroptosis. Besides, HCG18 was involved in the responsiveness of HCCs to sorafenib treatment, and knockdown of HCG18 can promote the sensitivity of HCC cells to sorafenib, inferring that targeting HCG18 might be an effective strategy to overcome sorafenib resistance in HCC 51. Recent research findings have provided confirmation that the induction of cellular ferroptosis can greatly enhance the responsiveness of tumors to sorafenib, underscoring the crucial role of ferroptosis in addressing patients who are resistant to sorafenib treatment 52.

Together, in this paper, we identified miR-30a-5p as the only DEMi associated with iron death in HCC using multiple specialized databases such as GSE69164, GSE77509, TCGA and other screening; relevant target lncRNAs were obtained from Inbase and starBase databases; on this basis, survival analysis was performed, and it was finally determined that HCG18 or RRM2 high-expression HCC patients had a poorer prognosis, and miR-30a-5p high-expression HCC patients had a longer survival, linking HCC to ferroptosis. Additionally, HCC is an aggressive malignant tumor that is difficult to diagnose at early stage and lacks effective treatment strategies, which poses a significant health risk to the public. In this context, the present study provides new theoretical support and practical guidance for the creation of new biomarkers and therapeutic targets for HCC, which has important translational research value and potential application prospects.

However, the present study has some limitations. Firstly, due to the limitations of the experimental period and conditions, there are still deficiencies in this experiment. In this study, we mainly relied on the subcutaneous tumor model of nude mice to verify the relevant conclusions. If we can further construct the "human liver cancer nude mouse subcutaneous transplantation tumor" model or HCG18 knockout mouse model on the basis of this model, the results of this study will be more convincing. Additionally, the number of clinical samples collected was limited due to constraints such as time and the number of cancer patients. We also considered potential explanations for the differential expression patterns of HCG18 and RRM2 observed between stage III and stage IV in Figures 2b, 2e, and 2f. These differences may arise from sample heterogeneity. Specifically, the TCGA dataset incorporates multi-center global cohorts that inherently contain confounding variables (e.g., molecular subtype variations, differences in prior therapeutic interventions), whereas our clinical cohort likely reflects region-specific or population-specific characteristics. As the result, Samples from relevant patients will continue to be collected for sample size expansion and analysis. Finally, our study lacks direct evidence that HCG18 affects drug sensitivity in HCC, which will also be the focus of our future studies.

## 5. Conclusions

Our study has discovered a novel regulatory pathway involving HCG18/miR-30a-5p/RRM2/GSS in HCC (Fig. 9m). Directly targeting this pathway enhances cell sensitivity to ferroptosis and provides a potential approach to mitigate drug resistance of HCC patients. HCG18 confers resistance to ferroptosis and holds promise as a potential target for ferroptosis-dependent therapy.

## Supplementary Material

Supplementary figures and tables.
