# Supplementary material for: Targeting HCG18 counteracts ferroptosis resistance via blocking the miR-30a-5p/RRM2/GSS pathway in hepatocellular carcinoma
Source: Int J Biol Sci. 2025 Mar 21;21(6):2550–67. doi: 10.7150/ijbs.104127 (PMC12035896; doi:10.7150/ijbs.104127)
Supplement: Supplementary file 1 — Supplementary figures and tables. [file ijbsv21p2550s1.pdf]

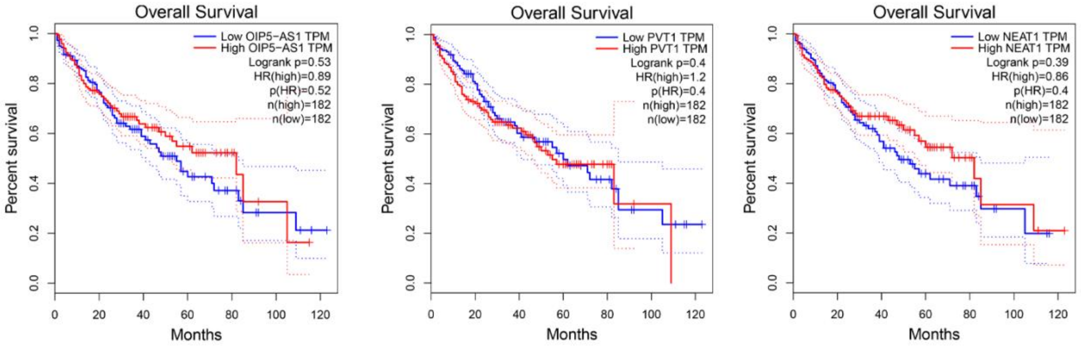

Supplementary Fig. 1: Kaplan-Meier survival analysis of OIP5-AS1, PVT1 and NEAT1 in HCC.

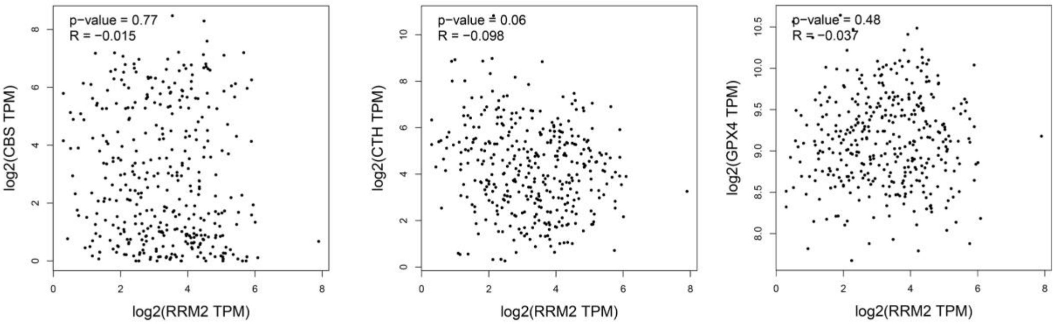

Supplementary Fig. 2: TCGA database was used to analyze the correlation of CBS-RRM2, CTH-RRM2, and GPX4-RRM2 in HCC.

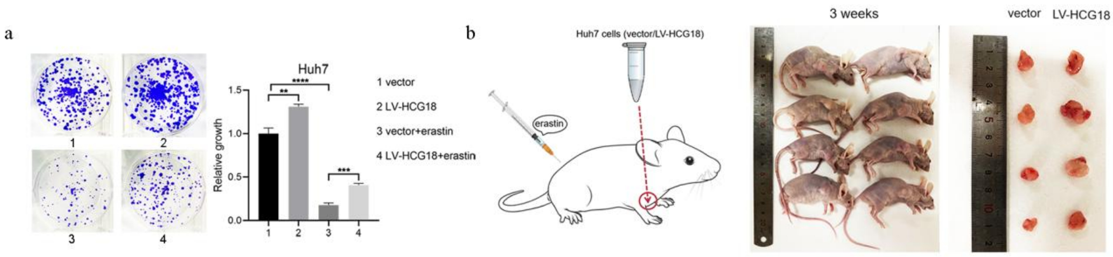

Supplementary Fig. 3: **a** The colony formation assay was used to measure the colony formation ability of Huh7 cells transfected with indicated constructs. **b** (Left)Schematic diagram of animal experiment. (Right) Comparison of subcutaneous tumor size in nude mice after injecting Huh7 cells stably transfected with vector or LV-HCG18.

Supplementary Table 1. Clinical and survival data of 150 HCC patients

samples	TNM stage	Time(months)	fustate	gender	Age
S201819122	IA(T1a,N0,cM0)	61	0	male	55
S201819725	IA(T1a,N0,cM0)	60	0	female	62
S201909283	IA(T1a,N0,cM0)	54	0	male	64
S202007324	IA(T1a,N0,cM0)	43	0	male	65
S202102480	IA(T1a,N0,cM0)	35	0	male	81
S202104823	IA(T1a,N0,cM0)	32	0	male	72
S202110736	IA(T1a,N0,cM0)	31	0	male	70
S202107184	IA(T1a,N0,cM0)	29	0	female	49
S202115747	IA(T1a,N0,cM0)	30	0	male	71
S201906430	II(T2,N0,cM0)	36	1	male	37
S202200928	IA(T1a,N0,cM0)	22	0	male	58
S201906995	II(T2,N0,cM0)	36	1	male	52
S202204458	IA(T1a,N0,cM0)	21	0	male	55
S202208718	IA(T1a,N0,cM0)	19	0	male	37
S201807303	IB(T1b,n0,cM0)	66	0	male	50
S202002602	II(T2,N0,cM0)	24	1	male	68
S201812720	IB(T1b,n0,cM0)	64	0	female	55
S201815539	IB(T1b,n0,cM0)	12	1	male	52
S201813875	IB(T1b,n0,cM0)	64	0	male	80
S201817363	IB(T1b,n0,cM0)	62	0	male	61
S201814803	IB(T1b,n0,cM0)	63	0	male	50
S201818534	IB(T1b,n0,cM0)	62	0	male	59
S201819267	IB(T1b,n0,cM0)	61	0	male	57
S202104896	II(T2,N0,cM0)	24	1	male	68
S201820097	IB(T1b,n0,cM0)	60	0	male	60
S201820260	IB(T1b,n0,cM0)	60	0	male	67
S201903442	IB(T1b,n0,cM0)	57	0	male	51

S201904085	IB(T1b,n0,cM0)	57	0	male	40
S201903918	IB(T1b,n0,cM0)	57	0	male	51
S201903748	IB(T1b,n0,cM0)	57	0	male	49
S201904661	IB(T1b,n0,cM0)	56	0	male	49
S201903030	IB(T1b,n0,cM0)	56	0	male	50
S201910886	IB(T1b,n0,cM0)	54	0	female	63
S201911472	IB(T1b,n0,cM0)	54	0	female	70
S201912317	IB(T1b,n0,cM0)	53	0	male	66
S201913092	IB(T1b,n0,cM0)	45	1	male	70
S201912925	IB(T1b,n0,cM0)	52	0	male	63
S202002235	IB(T1b,n0,cM0)	45	0	male	47
S202002242	IIIA(T3,N0,cM0)	18	1	male	48
S202002259	IB(T1b,n0,cM0)	44	0	male	57
S202002383	IB(T1b,n0,cM0)	44	0	male	50
S202003557	IB(T1b,n0,cM0)	41	0	male	66
S202006463	IB(T1b,n0,cM0)	40	0	male	48
S202003528	IB(T1b,n0,cM0)	42	0	male	67
S202003427	IB(T1b,n0,cM0)	41	0	male	71
S202007167	IB(T1b,n0,cM0)	40	0	male	50
S202007749	IB(T1b,n0,cM0)	40	0	male	47
S202010786	IB(T1b,n0,cM0)	39	0	male	51
S202011411	IB(T1b,n0,cM0)	38	0	male	50
S202012045	IB(T1b,n0,cM0)	38	0	male	50
S202011963	IB(T1b,n0,cM0)	37	0	male	49
S202104493	IB(T1b,n0,cM0)	32	0	male	68
S202106039	IB(T1b,n0,cM0)	32	0	male	51
S202104442	IB(T1b,n0,cM0)	33	0	male	61
S202110302	IB(T1b,n0,cM0)	30	0	male	80
S202109989	IB(T1b,n0,cM0)	30	0	male	72

S202111361	IB(T1b,n0,cM0)	31	0	male	64
S202116155	IB(T1b,n0,cM0)	30	0	female	81
S202110490	IB(T1b,n0,cM0)	30	0	male	66
S202110788	IB(T1b,n0,cM0)	31	0	male	64
S202113912	IB(T1b,n0,cM0)	29	0	male	58
S202119856	IB(T1b,n0,cM0)	29	0	male	60
S202201246	IB(T1b,n0,cM0)	22	0	male	53
S202202988	IB(T1b,n0,cM0)	23	0	male	59
S202202629	IB(T1b,n0,cM0)	22	0	male	60
S202203234	IB(T1b,n0,cM0)	21	0	male	68
S202205830	IB(T1b,n0,cM0)	20	0	male	76
S202206700	IB(T1b,n0,cM0)	20	0	male	56
S202206785	IB(T1b,n0,cM0)	20	0	male	37
S202207635	IB(T1b,n0,cM0)	19	0	male	57
S202209294	IB(T1b,n0,cM0)	18	0	male	47
S202210506	IB(T1b,n0,cM0)	18	0	male	54
S202210380	IB(T1b,n0,cM0)	18	0	male	53
S201813966	II(T2,N0,cM0)	63	0	male	66
S201815873	II(T2,N0,cM0)	12	1	male	59
S201813662	II(T2,N0,cM0)	64	0	female	71
S201814228	II(T2,N0,cM0)	5	1	male	52
S201814282	II(T2,N0,cM0)	64	0	male	46
S201817001	II(T2,N0,cM0)	63	0	female	62
S201816399	II(T2,N0,cM0)	18	1	male	65
S201819006	II(T2,N0,cM0)	36	1	male	65
S201819936	II(T2,N0,cM0)	60	0	male	53
S201820145	II(T2,N0,cM0)	12	1	male	67
S201818855	II(T2,N0,cM0)	61	0	male	56
S201818473	II(T2,N0,cM0)	61	0	female	63

S201820477	II(T2,N0,cM0)	24	1	male	57
S201826505	II(T2,N0,cM0)	59	0	male	78
S201901598	II(T2,N0,cM0)	58	0	male	59
S201903311	II(T2,N0,cM0)	57	0	male	49
S201904615	II(T2,N0,cM0)	24	1	male	46
S201905624	II(T2,N0,cM0)	55	0	male	50
S201906581	II(T2,N0,cM0)	55	0	female	63
S201910426	II(T2,N0,cM0)	54	0	male	75
S201910882	II(T2,N0,cM0)	54	0	male	74
S201911108	II(T2,N0,cM0)	54	0	male	51
S201907124	II(T2,N0,cM0)	55	0	male	66
S201912085	II(T2,N0,cM0)	54	0	male	61
S201912482	II(T2,N0,cM0)	53	0	male	57
S201912924	II(T2,N0,cM0)	52	0	male	64
S202002567	II(T2,N0,cM0)	42	0	male	63
S202001253	II(T2,N0,cM0)	45	0	male	46
S202007510	II(T2,N0,cM0)	40	0	female	68
S202003905	II(T2,N0,cM0)	41	0	male	63
S202005870	II(T2,N0,cM0)	41	0	male	64
S202009103	II(T2,N0,cM0)	39	0	male	43
S202009756	II(T2,N0,cM0)	38	0	male	52
S202012041	II(T2,N0,cM0)	38	0	male	54
S202104250	II(T2,N0,cM0)	34	0	male	56
S202108795	II(T2,N0,cM0)	31	0	male	53
S202109430	II(T2,N0,cM0)	31	0	male	64
S202109174	II(T2,N0,cM0)	30	0	male	56
S202111365	II(T2,N0,cM0)	31	0	male	65
S202107799	II(T2,N0,cM0)	32	0	male	66
S202111494	II(T2,N0,cM0)	26	1	male	80

S202114872	II(T2,N0,cM0)	30	0	male	59
S202109900	II(T2,N0,cM0)	31	0	male	52
S202200484	II(T2,N0,cM0)	23	0	male	51
S202201355	II(T2,N0,cM0)	19	0	female	53
S202202916	II(T2,N0,cM0)	21	0	male	47
S202203319	II(T2,N0,cM0)	22	0	male	60
S202204163	II(T2,N0,cM0)	21	0	male	70
S202206513	II(T2,N0,cM0)	20	0	male	66
S202209880	II(T2,N0,cM0)	18	0	male	68
S201816212	IIIA(T3,N0,cM0)	62	0	male	62
S201815809	IIIA(T3,N0,cM0)	63	0	male	52
S201821027	IIIA(T3,N0,cM0)	8	1	male	65
S201823667	IIIA(T3,N0,cM0)	12	1	male	52
S201902042	IIIA(T3,N0,cM0)	10	1	male	64
S201906373	IIIA(T3,N0,cM0)	18	1	male	43
S202201771	IA(T1a,N0,cM0)	19	0	female	72
S201910260	IIIA(T3,N0,cM0)	30	1	female	59
S201912088	IIIA(T3,N0,cM0)	6	1	female	70
S202001782	IIIA(T3,N0,cM0)	12	1	male	52
S202005569	IIIA(T3,N0,cM0)	18	1	male	34
S202204155	IA(T1a,N0,cM0)	21	0	male	55
S202101178	IIIA(T3,N0,cM0)	18	1	female	59
S202107910	IIIA(T3,N0,cM0)	29	0	male	74
S202117923	IIIA(T3,N0,cM0)	24	1	male	69
S202203624	IIIA(T3,N0,cM0)	12	1	female	54
S202207597	IIIA(T3,N0,cM0)	19	0	male	61
S202204850	IA(T1a,N0,cM0)	21	0	male	74
S202002054	IIIB(T4,N0,cM0)	8	1	male	57
S202009373	IIIB(T4,N0,cM0)	6	1	female	50

S202010615	IIIB(T4,N0,cM0)	12	1	male	67
S202111250	IIIB(T4,N0,cM0)	18	1	male	79
S202200003	IIIB(T4,N0,cM0)	8	1	male	57
S202203816	IIIB(T4,N0,cM0)	12	1	male	57
S202204235	IIIB(T4,N0,cM0)	21	0	male	54
S202201176	IV(T2,N1c,cM0)	4	1	male	32
S202010929	IV(T4,N1,cM0)	6	1	male	73

Supplementary Table 2. siRNA and primer sequences

name	sequence (5' → 3')
siHCG18 sense	AGCUGAAAGUCGACGAAGA
siHCG18 anti-sense	UCUUCGUCGACUUUCAGCU
siRRM2 sense	CACCGGATCCTCCTCGCGGTCTTGC
siRRM2 anti-sense	AAACGCAAGACCGCGAGGAGGATCC
HCG18 Forward Primer	ATTCTCACTCTGGGGTTGGG
HCG18 Reverse Primer	TGATGTTGGCTGTGGGTTTG
RRM2 Forward Primer	CACGGAGCCGAAAACTAAAGC
RRM2 Reverse Primer	TCTGCCTTCTTATACATCTGCCA
miR-30a-5p Forward Primer	CGGGCTGTAAACATCCTCGA
miR-30a-5p Reverse Primer	CAGCCACAAAAGAGCACAAT
GAPDH Forward Primer	CATGAGAAGTATGACAACAGCCT
GAPDH Reverse Primer	AGTCCTTCCACGATACCAAAGT
U6 Forward Primer	CTCGCTTCGGCAGCACA
U6 Reverse Primer	AACGCTTCACGAATTTGCGT

Supplementary Table 3. Antibodies used in this study

Antibodies	Source	Product No.	Dilution
RRM2	Proteintech	67006-1-Ig	1:1000 (IHC)<br>1:500 (IF)
RRM2	Abcam	ab172476	1:5000 (WB)
Ki67	Abcam	ab92742	1: 1000 (IHC)
FLAG	sigma	F7425	1:5000 (IP)
HA	sigma	H6908	1:5000 (IP)
GSS	Proteintech	15712-1-AP	1:200 (IF)
GSS	Santa Cruz	sc-166882	1:500 (IP)
GSS	Abcam	ab124811	1: 5000 (WB)
GADPH	Abcam	ab8245	1: 1000 (WB)
